# Bundled-Optode Method in Functional Near-Infrared Spectroscopy

**DOI:** 10.1371/journal.pone.0165146

**Published:** 2016-10-27

**Authors:** Hoang-Dung Nguyen, Keum-Shik Hong, Yong-Il Shin

**Affiliations:** 1 Department of Cogno-Mechatronics Engineering, Pusan National University, 2 Busandaehak-ro, Geumjeong-gu, Busan, 46241, Republic of Korea; 2 School of Mechanical Engineering, Pusan National University, 2 Busandaehak-ro, Geumjeong-gu, Busan, 46241, Republic of Korea; 3 Department of Rehabilitation Medicine, School of Medicine, Pusan National University & Research Institute for Convergence of Biomedical Science and Technology, Pusan National University Yangsan Hospital, 20, Geumo-ro, Mulgeum-eup, Yangsan-si, Gyeongsangnam-do, 50612, Republic of Korea; Tokai University, JAPAN

## Abstract

In this paper, a theory for detection of the absolute concentrations of oxy-hemoglobin (HbO) and deoxy-hemoglobin (HbR) from hemodynamic responses using a bundled-optode configuration in functional near-infrared spectroscopy (fNIRS) is proposed. The proposed method is then applied to the identification of two fingers (i.e., little and thumb) during their flexion and extension. This experiment involves a continuous-wave-type dual-wavelength (760 and 830 nm) fNIRS and five healthy male subjects. The active brain locations of two finger movements are identified based on the analysis of the *t*- and *p*-values of the averaged HbOs, which are quite distinctive. Our experimental results, furthermore, revealed that the hemodynamic responses of two-finger movements are different: The mean, peak, and time-to-peak of little finger movements are higher than those of thumb movements. It is noteworthy that the developed method can be extended to 3-dimensional fNIRS imaging.

## Introduction

The objective of this paper is to characterize specific brain regions in the left motor cortex when finger-movements (i.e., flexion and extension) of the right little and thumb fingers are made. For this, the hemodynamic responses (HRs) upon finger movements are measured using functional near-infrared spectroscopy (fNIRS) with a bundled-optode configuration. The activated brain regions (upon 48 channel data) associated with individual finger movements are identified based on *t*- and *p*-value analyses of the averaged oxy-hemoglobin (HbO) concentration over five trials.

In the field of fNIRS-based brain-imaging, three techniques are used: continuous-wave (CW), frequency-domain (FD), and time-domain (TD). First, CW-fNIRS, which utilizes constant-intensity light sources of 650~1,000 nm wavelength, is the most popular technique. It measures the scattered light through the brain using light-detectors (optodes) positioned 2~4 cm apart from the light-source on the scalp. Typically, the changes of the hemoglobin concentration can be computed, based on the modified Beer-Lambert law (MBLL), according to the known extinction coefficients and differential path-length factor [1–14]. Recently, this technique has been integrated into portable equipment using light emitting diodes, electronic circuits, and wireless transceivers [8,15]. Second, FD-fNIRS is a complex technique: It proceeds by modulation of the amplitude of a light source at a very high frequency (i.e., hundreds of megahertz) [16–19]. It then measures the absolute values of the HbO and deoxy-hemoglobin (HbR) concentrations based on the changes in the amplitudes and phases of the detected light intensities. The main advantage of the FD approach is the measurement of the absolute values of hemoglobin concentrations. Finally, the TD spectroscopy is an expensive and intricate technique [20,21] that utilizes a very short (on the order of picoseconds) pulsed light to illuminate a specific brain region. This technique allows the measurement of the mean optical path length, absorption coefficient, and scattering coefficient based on the detected back-reflected light. By application of the measured quantitative coefficients, the absolute values of hemoglobin concentrations can then be computed.

The spatially resolved spectroscopy (SRS) approach [6,22–25] was used to measure optical properties of tissues. In this approach, one emitter and at least two arranged detectors are used, which means that at least two channels are utilized. The absorption coefficient can then be computed based on the absorbance gradient with respect to the emitter-detector distance. One of the early studies employing this method is Farrell et al. [26]. They measured the diffuse reflectance of light intensities by the distance between an emitter-detector pair (range: 1.0~10.0 mm). Then, from the phantom and muscle tissue data, they estimated the absorption and scattering coefficients using the nonlinear least square fitting approach. Based on the results, the absolute concentration values can be determined by way of the MBLL. Several subsequent studies also focused on the estimation of such coefficients from the measured data of both brain and muscle tissues [20,22,24,27]. In the present work, the SRS method is used with a bundled-optode arrangement (i.e., multiple emitters and multiple detectors arranged side by side). It is noteworthy that short-separation detectors (i.e., 0.5 cm in this paper) as well as long-separation detectors (i.e., 1~4 cm) are used at the same time in our method [28,29]. The superficial noise (i.e., the trend in the scalp) [28–30] is reduced by subtraction of the acquired signals between the short-separation detectors from those between the long-separation detectors.

Recently, the HRs in various brain areas (namely, the motor, visual, and somatosensory cortices) have been investigated [31–33]. However, finding a specific brain region in a given cortex (e.g., a local region in the motor cortex for a specific finger movement) is difficult. Some previous works have revealed overlapping of the activated sites upon individual finger movements [34–38]. One of the early studies on single-finger movement is Schieber and Hibbard [38]. In their work, 10 subjects were asked to extend their right index and middle fingers simultaneously and separately. They found that a separate movement of the fingers was significantly more active than two-finger coincident movement. Moreover, the functional activity increased as the finger-movement rates grew faster [39]. Also, the brain region corresponding to finger extension was more active than that to flexion [40]. Penfield and Rasmussen [41] illustrated the corresponding positions of brain functions in the cerebral cortex (both in the somatosensory and motor areas) by means of an electrical stimulation method (i.e., electrodes placed directly on the patient’s cortices). Recently, the active regions of finger movements in the motor cortex have been located using the functional magnetic resonance imaging technique [36,37,42,43]. Some demonstrated thumb movement to be located in the lateral site and little finger movement in the medial site [35,42,44]. Lately, the fNIRS technique has been utilized to investigate the active locations of the little and thumb fingers in association with the primary somatosensory cortex [45]: In their work, vibro-tactile stimulation was applied to the little and thumb fingers of eight experimental participants. They found that, in five out of eight participants, the active locations of two fingers could be distinguished. Most notably, Yamada et al. [46] established a method for acquisition, by means of a multi-distance probe (one emitter and two detectors), of the brain-functional signals corresponding to left- and right-thumb/index finger tapping. Recently, the active locations of finger movements have been identified by using an invasive technique, that is, an electrocorticography array [47].

In this paper, we present a method for detection of the (absolute) concentrations of HbO and HbR using CW-fNIRS with a bundled-optode configuration (i.e., non-invasive optical methodology). Using the developed method, the HRs upon individual (right) finger movements will be characterized first and the activated brain regions in the (left) motor cortex will be identified. Finally, and perhaps significantly, it is shown that the active locations of the two finger movements are quite distinctive.

## Theory: Bundled-Optode

According to the CW-fNIRS technique, the incident light from an emitter will travel into brain tissue and reflect back to the scalp of the subject. Therefore, a portion of the incident light can be measured by a detector placed a few centimeters (2~4 cm) apart from the emitter. This phenomenon is described by the Beer-Lambert equation as follows.

$$I_{\text{out}}(t,\lambda )=I_{\text{in}}(t,\lambda )e^{-\mu (t,\lambda )\times l},$$ (1)

where *I*_in_(*t*, *λ*) is the intensity of the incident light of wavelength *λ* at time *t*, *I*_out_(*t*, *λ*) is the intensity of the detected light at time *t* (of the same wavelength *λ*), *l* indicates the distance between an emitter and a detector, and *μ*(*t*, *λ*) expresses the linear attenuation coefficient. Note that *μ*(*t*, *λ*) can be split into two parts [48]:

$$\mu (t,\lambda )=\mu_{\text{a}}(t,\lambda )+\mu_{\text{s}}(\lambda ),$$ (2)

where *μ*_a_(*t*, *λ*) and *μ*_s_(*λ*), respectively, stand for the absorption and scattering coefficients. In the present work, the scattering coefficient *μ*_s_(*λ*) is assumed constant throughout the brain region, whose value in this paper is used as follows, see [20].

$$\mu_{\text{s}}(\lambda )=\gamma_{1}(1-\gamma_{2}\lambda ),$$ (3)

where *γ*_1_ = 1.45 mm^-1^ and *γ*_2_ = 4.5×10^−4^ nm^-1^. Using (1), the absorbance of the light at the *i*-th channel, *A*^*i*^(*t*, *λ*), is computed as follows.

$$A^{i}(t,\lambda )=-\ln\frac{I_{\text{out}}^{i}(t,\lambda )}{I_{\text{in}}^{i}(t,\lambda )}=\mu (t,\lambda )\times l^{i},$$ (4)

where the superscript *i* denotes the channel number, $I_{\text{in}}^{i}(t,\lambda )$ and $I_{\text{out}}^{i}(t,\lambda )$ are the intensities of the incident and detected lights at the *i*-th channel, respectively, and *l*^*i*^ indicates the distance between the emitter and the detector at the *i*-th channel.

**Fig 1:**
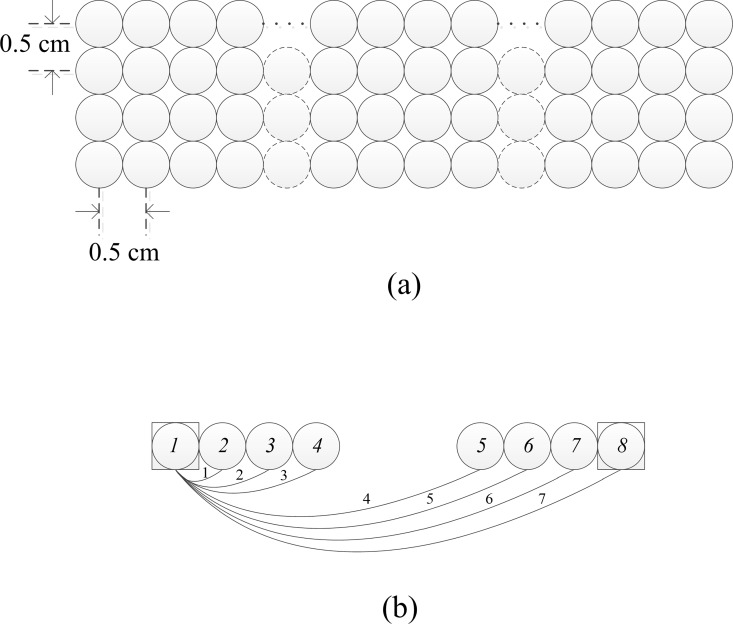
Bundled-optode arrangement. **(**a) Concept of bundled-optode arrangement; (b) 1-emitter and 7-detector configuration for HbO/HbR measurement (see Fig 2 for their assignments): Optode *1* (square) is emitter and optode *2* and optodes *3*~*8* are short- and long-separation detectors (circles), respectively.

**Fig 2:**
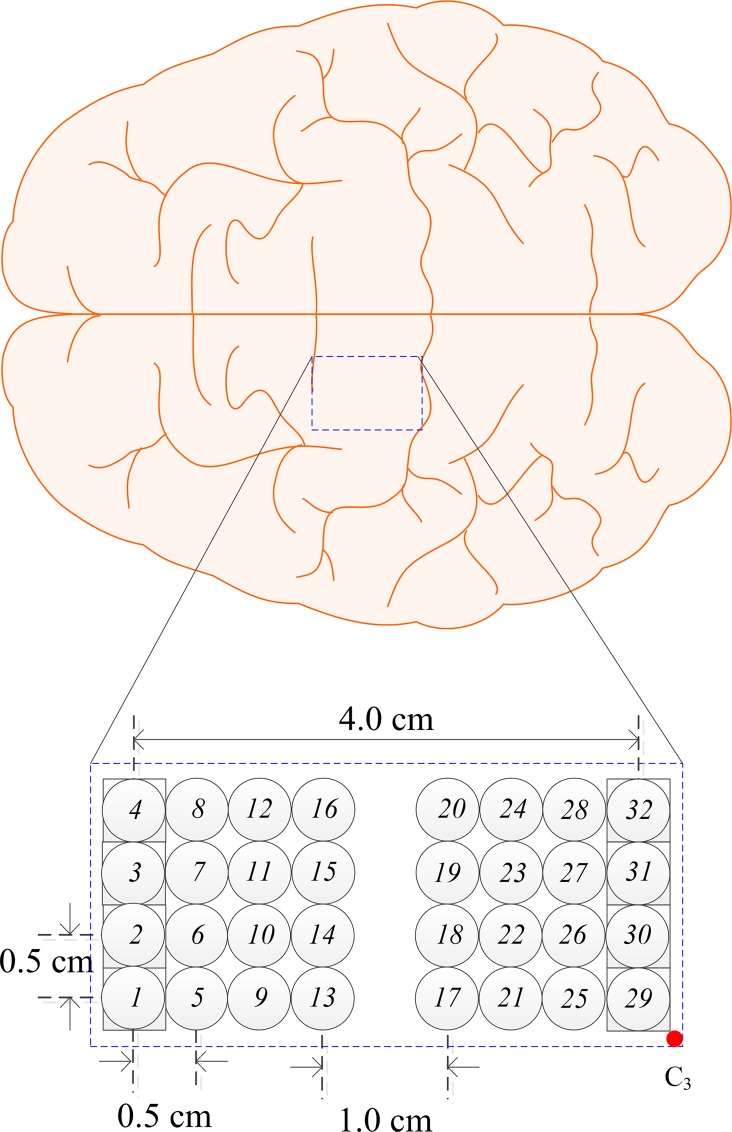
Optode arrangement on the left motor cortex for detection of right-finger movements. Squares and circles are emitters and detectors, respectively, and C_3_ denotes the reference point from the International 10–20 System.

Fig 1 illustrates the concept of the bundled-optode method for measurement of absolute HbO/HbR concentrations. Let the italic integer numbers (i.e., *1*, *2*, *3*, *4*, *5*, *6*, *7* and *8*) denote the locations of emitters (square) and detectors (circles), and let the plain integer numbers represent the channels formed by the emitter/detector pairs. A short-separation channel is formed from an emitter and a shortly separated detector (i.e., 0.5 cm) whereas long-separation channels are established from an emitter and detectors separated apart (i.e., 1~4 cm). For instance, consider the configuration of one emitter and seven detectors shown in Fig 1(B). It is noted that, from optode *1*, optode *2* is separated shortly and optodes *3*, *4*, *5*, *6*, *7*, and *8* are separated apart. Hence, one short-separation channel (i.e., emitter *1* and detector *2* or, reversely, emitter *8* and detector *7*) and six long-separation channels are made (i.e., emitter *1* and six detectors *3*~*8*). In the present work, a 32-optode-configuration operating at a sampling rate of 1.81 Hz was used (see Fig 2). In this case, the time difference between two neighboring channels is 17 msec (i.e., 1/(1.81 × 32)). Therefore, it can be assumed that the linear attenuation coefficient in (4), *μ*(*t*, *λ*), is almost the same in the neighboring channels (for instance, Chs. 1~7).

Then, for two neighboring channels in a local region, the absorbance difference can be defined as

$$\delta A^{ij}(t,\lambda )=A^{j}(t,\lambda )-A^{i}(t,\lambda )=\mu (t,\lambda )\left|l^{j}-l^{i}\right|,$$ (5)

where *i =* 1, *j* = 2~7. Now, let

$$\delta l^{ij}=\left|l^{j}-l^{i}\right|$$ (6)

be the spatial distance between two neighboring channels *i* and *j*. Then, from (2) and (5)-(6), the linear attenuation coefficient can be approximated by the spatial absorbance gradient between two neighboring channels (i.e., *δA*^*ij*^(*t*,*λ*)/*δl*^*ij*^) as follows.

$$\begin{array}{l} \frac{\delta A^{ij}(t,\lambda )}{\delta l^{ij}}≅\mu_{\text{a}}(t,\lambda )+\mu_{\text{s}}(\lambda )\\ \;=a_{\text{HbO}}(\lambda )C_{\text{HbO}}(t)+a_{\text{HbR}}(\lambda )C_{\text{HbR}}(t)+\mu_{\text{s}}(\lambda ), \end{array}$$ (7)

where *a*_HbO_(*λ*) and *a*_HbR_(*λ*) are the extinction coefficients of HbO and HbR at wavelength *λ*, respectively, and *C*_HbO_(*t*) and *C*_HbR_(*t*) represent the concentrations of HbO and HbR, respectively. The configuration of one emitter and seven detectors in Fig 1(B), therefore, will make six absorbance gradients.

Finally, for two wavelengths, the following two absorbance spatial gradient equations are obtained.

$$\frac{\delta A^{ij}(t,\lambda_{1})}{\delta l^{ij}}=a_{\text{HbO}}(\lambda_{1})C_{\text{HbO}}(t)+a_{\text{HbR}}(\lambda_{1})C_{\text{HbR}}(t)+\mu_{\text{s}}(\lambda_{1}),$$ (8)

$$\frac{\delta A^{ij}(t,\lambda_{2})}{\delta l^{ij}}=a_{\text{HbO}}(\lambda_{2})C_{\text{HbO}}(t)+a_{\text{HbR}}(\lambda_{2})C_{\text{HbR}}(t)+\mu_{\text{s}}(\lambda_{2}).$$ (9)

Solving for *C*_HbO_(*t*) and *C*_HbR_(*t*), the (absolute) concentration values of HbO and HbR from a pair of *i* and *j* channels (i.e., one short-separation channel and one long-separation channel) in a local region can be obtained as follows:

$${\left[\begin{array}{c} C_{\text{HbO}}(t)\\ C_{\text{HbR}}(t) \end{array}\right]}_{ij}={\left[\begin{array}{cc} a_{\text{HbO}}(\lambda_{1}) & a_{\text{HbR}}(\lambda_{1})\\ a_{\text{HbO}}(\lambda_{2}) & a_{\text{HbR}}(\lambda_{2}) \end{array}\right]}^{-1}\left[\begin{array}{c} \frac{\delta A^{ij}(t,\lambda_{1})}{\delta l^{ij}}-\mu_{\text{s}}(\lambda_{1})\\ \frac{\delta A^{ij}(t,\lambda_{2})}{\delta l^{ij}}-\mu_{\text{s}}(\lambda_{2}) \end{array}\right].$$ (10)

## Method

### Ethics Statement

This work was approved by the Institutional Review Board of Pusan National University. Written consents were obtained from all subjects prior to the start. All experiments were conducted in accordance with the ethical standards encoded in the latest Declaration of Helsinki.

### Equipment

A dual-wavelength (760 and 830 nm) CW-fNIRS instrument (DYNOT; NIRx Medical Technologies, USA) was utilized to acquire the brain signals generated by the movements of two fingers (little, thumb) of the right hand. Forty-eight (48) channels in the left motor cortex at a sampling rate of 1.81 Hz were configured (see Fig 2).

### Subjects and Experimental Design

Five healthy male subjects (mean age 36.2; range: 33~37 years) with shaved heads were invited to perform experiments (little and thumb finger movements) involving the left motor cortex. All of the subjects are normal. Four of them are right handed. None of them had any neurological impairment or mental disorder. Prior to the experiments, the nature of the experiment was clearly explained to the subjects. To eliminate any interference from noise, the experiments were conducted in a dark and quiet room. The subjects were asked to sit freely on a comfortable chair and not to move their body during the experiment. Before starting, the subjects were carefully trained how to move their fingers.

**Fig 3:**
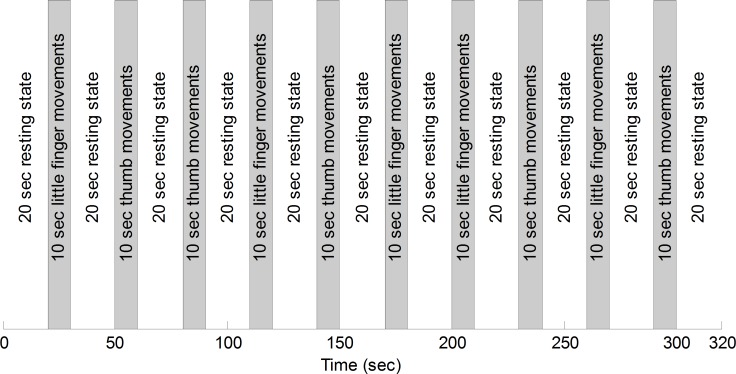
The experimental paradigm for ten trials of right finger movements (i.e., one trial is 10 sec flexion/extension of a finger followed by 20 sec rest). The five trials of little/thumb finger movements are randomly performed after the initial 20 sec rest.

Fig 3 describes the experimental paradigm: One experiment comprises ten trials of little and thumb finger movements. One trial consists of a 10 sec task and a 20 sec rest. After a 20 sec initial rest before the first trial, each subject was asked to perform finger movements (i.e., flexion and extension) for 10 sec, which was randomly repeated five times for each finger. Therefore, one experiment took 320 sec. To increase the brain activity during the 10 sec task period, the subjects were asked to move their (right) little/thumb finger as fast as they could, without paying attention to the number of flexions/extensions, and to relax during the 20 sec rest period. A laptop computer with a fifteen-inch screen was utilized to display pictures indicating each finger. The distance between the subject’s eyes and the laptop screen was adjusted to approximately 60 cm, so that the subjects could clearly see the indicated finger. The subjects were also instructed to keep their eyes open during the experiments. During the rest period, a black screen was shown to relax the subjects’ eyes.

### Data Processing

The measured fNIRS data are the intensities of light, which are to be converted to the absolute concentrations of HbX (i.e., HbO and HbR) using (10) and our own Matlab (the Mathworks, Inc., USA) codes. First, a baseline-correction method is utilized to eliminate the drifting phenomena in the acquired fNIRS data [5,49]. In our work, a fourth order polynomial has been fit to the recorded HbX data of each channel, and the fitted curve has been subtracted from the measured HbX signal [50]. Second, the baseline-corrected HbX signals are filtered by a low-pass filter with a cut-off frequency of 0.15 Hz to remove any respiration and pulse-related physiological noises.

### Bundled-Optode Configuration

In the present work, a bundled-optode configuration is used to improve the spatial resolution, which can detect the brain activities in local brain regions. The configuration in Fig 2 consists of 8 emitters and 24 detectors resulting in 48 channels. As in Fig 1(B), a local brain region has been specified by six channels. In each local region, the six absolute HbX concentrations are measured using the developed method in the “Theory” section. The proposed method uses pairs of short- and long-separation channels to compute the absolute values of hemoglobin concentrations (see (10)), while the conventional MBLL computes the variations of HbX concentrations of a single channel.

### Contrast-to-Noise Ratio

Contrast-to-noise ratio (CNR) computes the difference of the means of the signals during the task and the rest [51–53]. To validate our proposed method, the CNR is used to investigate the signal to noise ratio from the sense that a high CNR value indicates a high ratio of the signal upon the task against that to noise. The CNR is computed as follows.

$$\text{CNR}=\frac{\text{mean}\left(task\right)-\text{mean}(rest)}{\sqrt{\mathrm{var}(task)+\mathrm{var}(rest)}},$$ (11)

where “task” indicates the task period and “rest” denotes the rest period before starting to move fingers. In the present work, the duration of 6~16 sec was assigned to “task” and that of -6~0 sec from the onset time was set to “rest”.

### Regions of Interest

The regions of interest (ROIs) are the brain regions consisting of the channels in which the *t*-values are higher than the critical *t*-value (*t*_crt_) [54]. In this work, the *t*_crt_ is set to 1.674 according to the degree of freedom (*M*– 1 = 53, *M* is number of data samples of a trial) and the significance level (i.e., *α* = 0.05 for one-tailed test). The *t*-value of the averaged HbO of each channel is computed using the “robustfit.m” function available in Matlab. With this function, the averaged HbO in each channel is compared to the expected HR model (or the designed HR) by the least squares method, in which the designed HR is produced by convolving a trial period (i.e., 10 sec task and 20 sec rest) with the canonical HR function (i.e., the difference of two gamma functions) [5,10,31]. Then, the *t*- and *p*-values are used to determine the active channels: The criteria for “active” are *t*-value > 0 and *p*-value < 0.05. If these criteria are not met, the channel is considered inactive.

### Local Active Brain Regions

**Fig 4:**
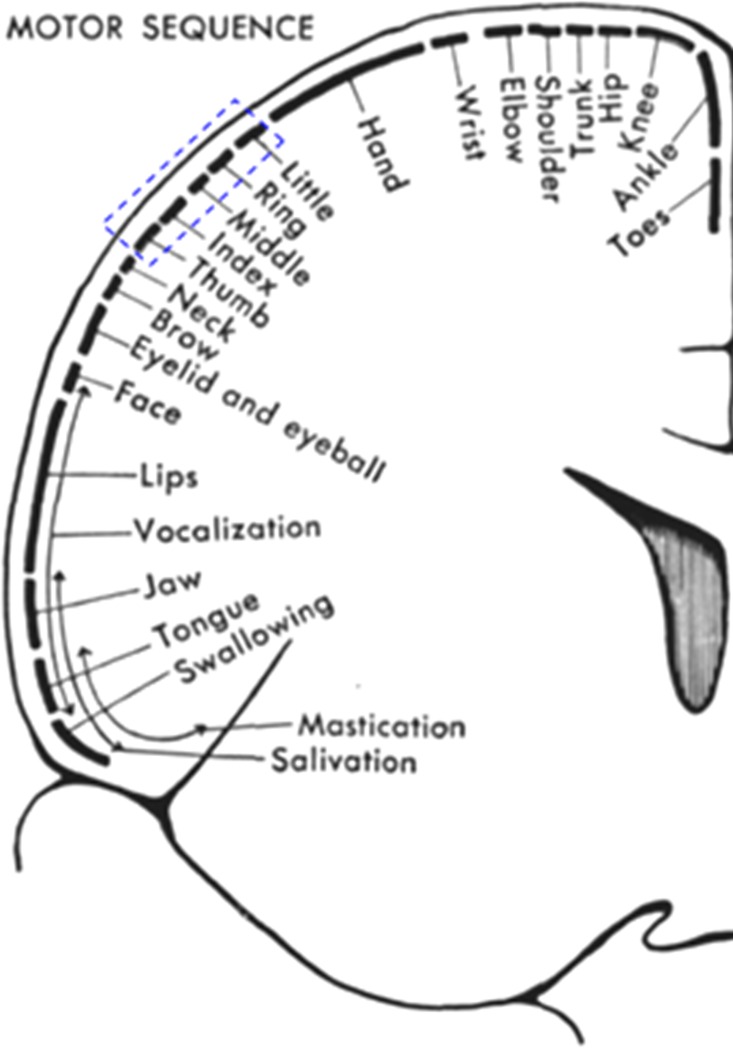
Brain regions of fingers directly detected by electrocorticogram in the motor cortex (blue dashed rectangle), see Fig 9 in [41].

**Fig 5:**
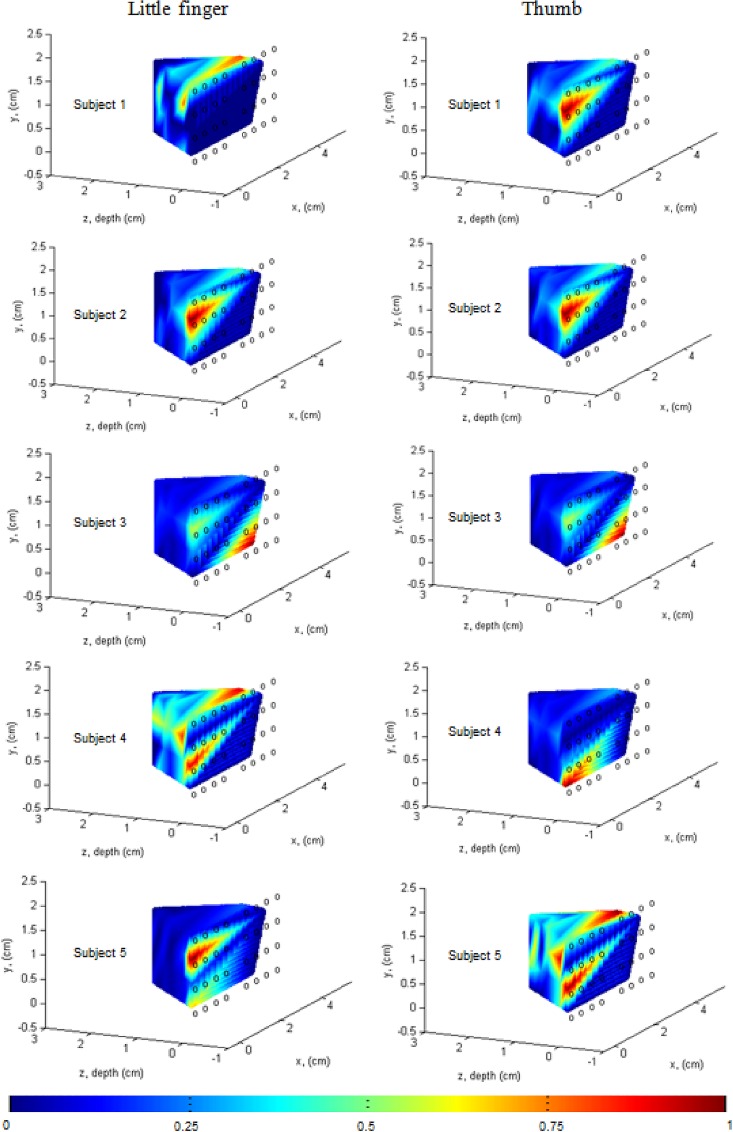
Local activation maps of little (left) and thumb (right) finger movements. The small circles indicate the optodes’ positions and the color bar at the bottom denotes the activation strength according to the normalized mean value between 0~1 range.

Our objective is to evaluate the brain activities in local brain regions. Fig 4 presents the brain regions related to fingers (blue dashed rectangle) established by electrocorticogram [41]. In our experiment, the movements of little and thumb fingers are randomly performed to enhance the activity strength in the corresponding brain regions [37]. For each experiment, 48 channels with 10 trials per channel (i.e., 48 × 10 = 480 total trials) are considered. The coordinate of each channel is calculated from the known probe geometry in Fig 2. To present the active locations in the three-dimensional (3D) space, the following steps are used: Step i) the HbOs in each channel are averaged across five trials of each finger; Step ii) if the *t*-value of the averaged HbO is greater than *t*_crt_ = 1.674 and the *p*-value is less than 0.05, the channel is identified as a channel in the ROI. Next, the mean value of the 4~14 sec window of the identified channel is computed. On the other hand, if the *t*-value of the channel is less than *t*_crt_ (i.e., the channel does not belong to the ROI), whose mean value is set to zero to make a clear 3D image, see Fig 5; Step iii) the mean values of 48 channels are normalized within the 0~1 range; Step iv) the normalized mean-value of the averaged HbO of each channel in the ROI is coded in color and plotted at its 3D coordinate using the “fscatter3.m” function available at https://www.mathworks.com/matlabcentral/fileexchange/2993-fscatter3-m. The 3D coordinates assignment procedure of the channels is described below. As seen in Fig 5, the highly active locations are identified with red colors (i.e., large mean values).

### Three-Dimensional Brain Map

In this work, the probe geometry is known (see Fig 2). Optode *1* is set to the reference coordinate. The coordinates of other optodes are computed based on optode *1*. The position of each channel in the 3D space (*x*, *y*, *z*) is calculated from the optodes’ positions. Let the positions of optode *1* (emitter) and optode *17* (detector) be (*x*_1_, *y*_1_, *z*_1_) and (*x*_2_, *y*_2_, *z*_2_), respectively. Then, the coordinate in the 3D space of the target point of this emitter-detector pair is computed as (*x*, *y*, *z*) = ((*x*_1_ + *x*_2_)/2, (*y*_1_ + *y*_2_)/2, sqrt((*x*_1_—*x*_2_)^2^ + (*y*_1_—*y*_2_)^2^)/2) [55,56]. In this case, the *z* coordinate indicates the depth in which the brain activity has occurred. According to the optode arrangement in Fig 2, the positions of 48 channels in the 3D space have been computed. The mean HbO value during the 4~14 sec window of each channel is plotted at its computed position with a coded color. Since the depths of channels are different, the active locations at different tissue layers can be detected [57]. Also, our proposed method can effectively remove the superficial noise from the scalp’s layer using (5). Since the banana shaped paths are closely located, it can give a local brain map with much improved spatial resolution [6,58].

## Results

**Fig 6:**
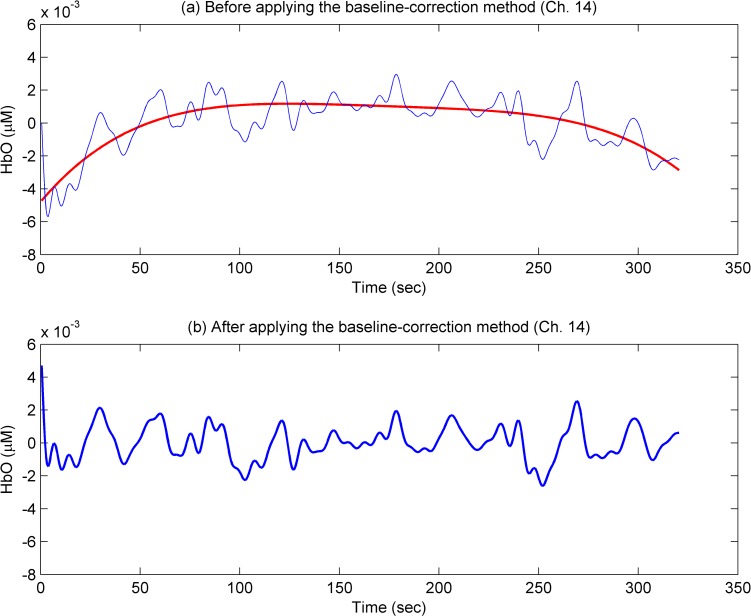
Illustration of the baseline-correction method (Ch. 14, Subject 5). (a) An HbO signal before applying the baseline-correction method (blue thin curve) and the fitted signal *f*(*x*) (red thick curve); (b) the corrected HbO signal (blue thick curve).

We developed a bundled-optode method to measure the concentrations of the HbX of little and thumb finger movements. In most cases, HbOs increased and HbRs decreased during the activation tasks. However, in some channels, HbRs also increased during the tasks. Since some early works [31,59,60] revealed that HbO is more sensitive and more reliable than HbR, HbO data have been mostly analyzed. In addition, some channels showed large baselines drifts [5,49]. In the present paper, a fourth order polynomial (i.e., *f*(*x*) = *a*_1_*x*^4^ + *a*_2_*x*^3^+ *a*_3_*x*^2^ + *a*_4_*x*+ *a*_5_) was fit to the recorded HbX in each channel [50]. To correct the drifting, the fitted curve has been subtracted from the measured HbX of the corresponding channel. Fig 6(A) shows the HbO (blue thin curve) of a representative channel (Ch. 14, Subject 5) before using the baseline-correction method and the fitted signal (red thick curve), *f*(*x*). Fig 6(B) depicts the corrected HbO signal (blue thick curve). As can be seen, the proposed baseline-correction method can effectively remove the baseline drifting in the fNIRS data.

**Fig 7:**
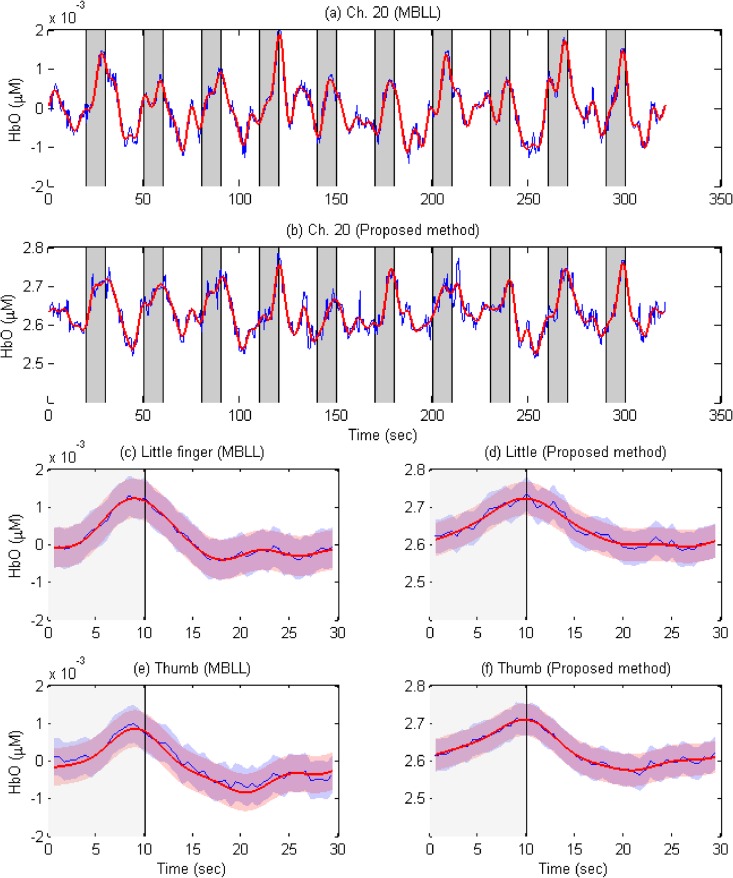
Comparison of the HbOs computed by the conventional MBLL method and the bundled-optode approach (Ch. 20, Subject 5, blue thin curves: raw data, red thick curves: low-passed filtered). (a) HbOs computed by the MBLL approach; (b) HbOs computed by the proposed method; (c)-(d) the averaged HbOs of the little finger movements (averaged over trials 1, 4, 6, 7, and 9) in (a)-(b), respectively; (e)-(f) the averaged HbOs of the thumb finger movements (averaged over trials 2, 3, 5, 8, and 10) in (a)-(b), respectively; the boundaries in (c)-(f) denote one standard deviation.

Fig 7 compares the HbOs computed by i) the conventional MBLL method (see Fig 7(A), 7(C) and 7(E)) and ii) the bundled-optode approach (see Fig 7(B), 7(D) and 7(F)) of an active channel (Ch. 20, Subject 5). The blue curves in Fig 7(A) and 7(B), respectively, are the HbO raw data obtained by the MBLL method and by Eq (10) (after the baseline correction was performed). The red thick curves in Fig 7(A) and 7(B), respectively, are the low-pass filtered HbOs from the blue curves. Gray bars in Fig 7(A) and 7(B) illustrate ten stimuli/trials of finger movements. Five trials (i.e., 1, 4, 6, 7, and 9) were assigned to little finger movements and other five trials (i.e., 2, 3, 5, 8, and 10) to thumb finger movements. Fig 7(C) and 7(E) plot the averaged HbOs of little and thumb finger movements, respectively, from Fig 7(A). Fig 7(D) and 7(F) show the averaged HbOs of little and thumb finger movements, respectively, from Fig 7(B). As can be seen, most HbOs increase during the 10 sec task period, and return to the baseline after the stimulation period is over. However, in a few trials (i.e., trials 1 and 7 in Fig 7(A)), it is seen that the HbO can begin to decrease prior to the end of the task: This can happen when the subject loses focus on the experiment.

**Fig 8:**
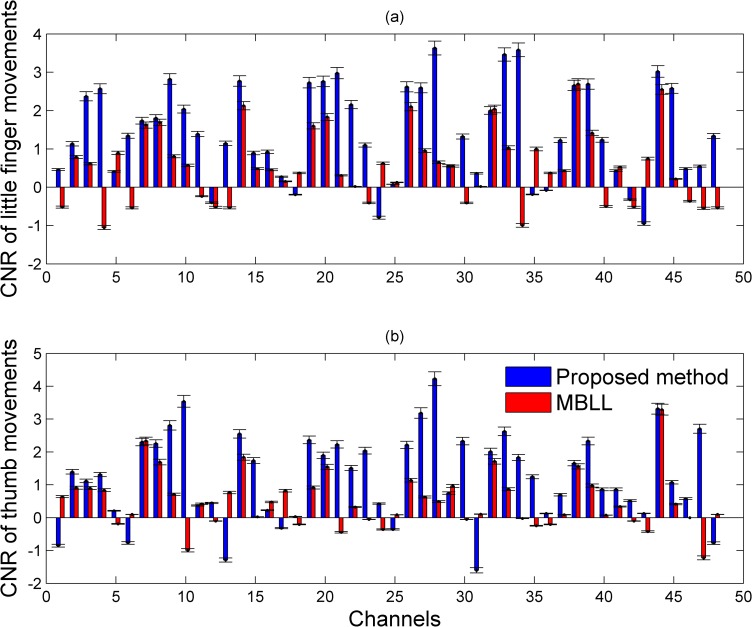
Comparison of the CNRs of HbOs in 48 channels (Subject 5). (a) The CNR of little movements; (b) the CNR of thumb movements: Red bars are the CNRs of HbOs computed by the conventional MBLL method whereas blue bars indicate the CNRs of those calculated by the proposed approach.

In our proposed method, a short-separation channel as well as long-separation channels is utilized to compute the absolute concentrations of HbOs. To evaluate the proposed method, the CNRs of HbOs were computed. Fig 8 plots the CNRs of HbOs of 48 channels of a representative subject (Subject 5) for little (Fig 8(A)) and thumb (Fig 8(B)) movements. Red bars denote the CNRs of the HbOs computed by the conventional MBLL method, whereas blue bars indicate the CNRs of those calculated by the proposed approach. As can be seen, the proposed method gives much higher CNRs than those using the conventional MBLL, which demonstrates that the proposed method can effectively remove noises from the scalp. It is interesting to observe that the CNRs of the proposed method in some channels are opposite to those obtained by the MBLL approach (see channels 1, 23, 30, and 47 in both Fig 8(A) and 8(B)), which typically happen in inactive channels. This can be explained by the fact that the noise, when using the proposed method, has been reduced (in both active and inactive cases).

**Fig 9:**
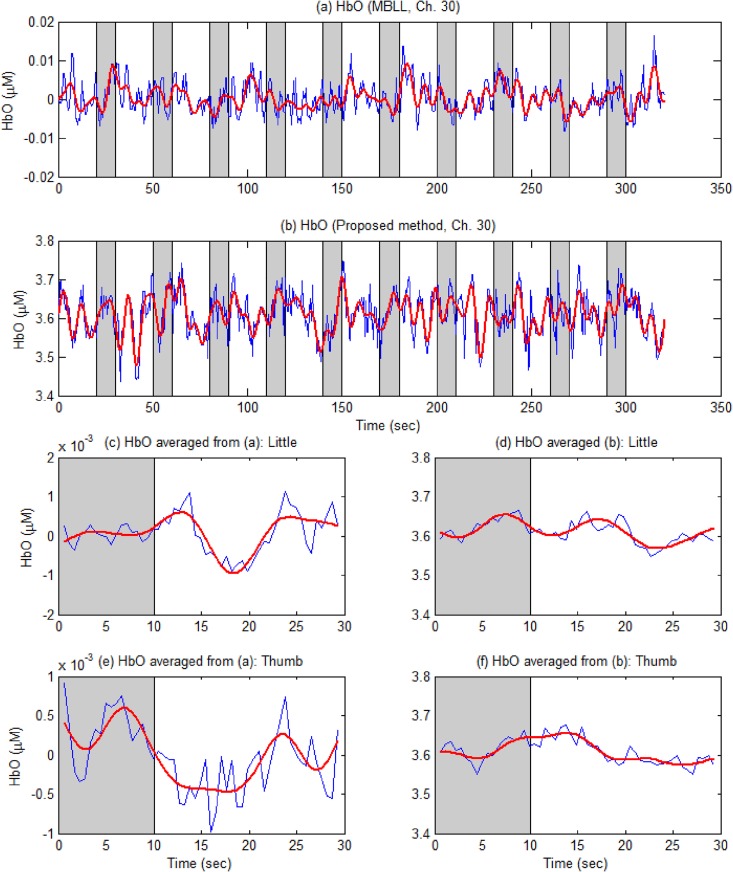
Comparison of two approaches for an inactive channel (Ch. 30, Subject 5). (a) HbOs computed by the MBLL approach (thin blue: raw, thick red: low-pass filtered); (b) HbOs by the proposed method; (c)-(d) the averaged HbO across 5 trials in (a) and (b), respectively, for little finger; (e)-(f) the averaged HbO for thumb finger.

To illustrate one advantage (noise reduction) of the proposed method, an inactive channel (Ch. 30) is considered. Fig 9(A) and 9(B) present the HbOs computed by the MBLL method and the proposed method, respectively. Like Fig 7(C) and 7(E), Fig 9(C) and 9(E) illustrate the averaged HbOs over 5 trials of little finger movements (i.e., 1, 4, 6, 7, and 9) and other 5 trials of thumb movements (i.e., 2, 3, 5, 8, and 10), respectively, from Fig 9(A). Fig 9(D) and 9(F) depict the averaged HbOs obtained by the proposed method. As seen in Fig 9(C) and 9(E), the HbOs obtained by the MBLL method are more fluctuating than those obtained by the proposed method (particularly in the resting state), which are due to noises. Recall that the mean HbO during the task is calculated between 6~16 sec and that during the rest is computed between -6 to the onset time. Typically, the mean during the task in Fig 9(C) is 2.20 × 10^−4^ μM and that during the rest is 4.71 × 10^−4^ μM (see the blue curve). Therefore, the CNR of the little finger movement task in Fig 9(C) is -0.41. Similarly, the mean during the task in Fig 9(E) is -4.47 × 10^−5^ μM while that during the rest is -1.19 × 10^−5^ μM (see the blue curve), therefore the CNR of the thumb movement task in Fig 9(E) becomes -0.05. However, if using the proposed method, the mean value during the task in Fig 9(D) is 3.63 μM and that during the rest is 3.59 μM. Therefore, the CNR becomes 1.32 for the little finger movement task in Fig 9(D). Similarly, the mean during the task in Fig 9(F) is 3.64 μM while that during the rest is 3.58 μM. Therefore, the CNR becomes 2.33. This explains why some CNR bar charts in Fig 8 are in opposite directions, and furthermore illustrates the noise removal capability of the proposed method.

**Fig 10:**
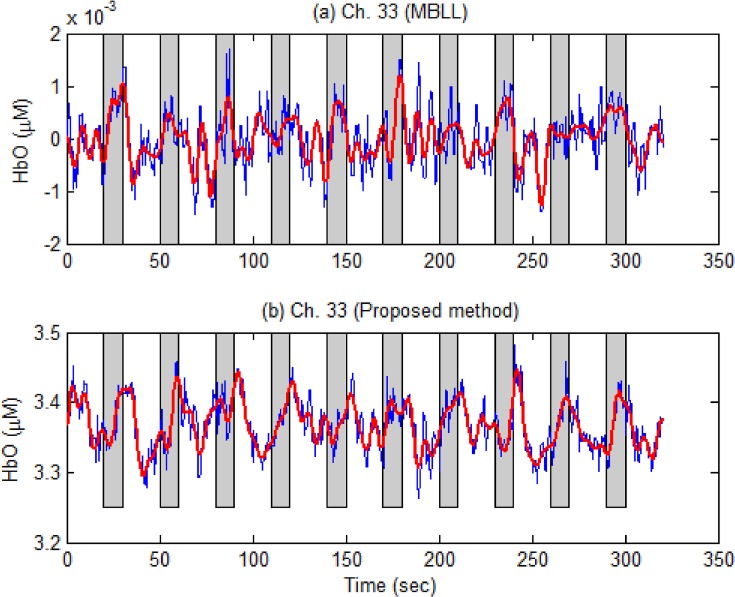
An example showing that the MBLL method cannot tell but the proposed method can tell (Ch. 33, Subject 5). (a) HbOs computed by the conventional MBLL method (blue thin curve: raw data, red thick curve: low-passed filtered); (b) HbOs obtained by the proposed method: In this case, the channel will be judged inactive by the MBLL method, while it will be judged active by the proposed method.

Additionally, to illustrate the capability of the proposed method, Fig 10 is provided (Ch. 33, Subject 5): Fig 10(A) depicts the HbOs produced by the MBLL method, whereas Fig 10(B) by the proposed method. As seen in Fig 10(A), the fluctuation of the HbO is not consistent with the given stimuli, whereas such fluctuation is very consistent in Fig 10(B). In this case, Ch. 33 would be judged inactive if the conventional MBLL method were used, whereas it would be judged active if the proposed method were used.

To locate the active territories upon right finger movements, the ROIs were first identified. That is, if the *t*-value of a channel was greater than *t*_crt_, the mean value of the averaged HbO of that channel was calculated. In contrast, if the *t*-value was not greater than *t*_crt_, its mean value was set to zero. Then, colors for the mean values were coded to plot a 3D image. The steps for locating the active brain regions were described in detail in the “Local active brain regions” section. Fig 5 presents the active locations of little and thumb finger movements of five subjects. Maps in the left side represent little finger movements, whereas those in the right side represent thumb movements. The small circles denote the optodes’ positions. The color bar at the bottom indicates the activation strength of the normalized mean value between 0~1 range. As can be seen, the active territories upon little and thumb finger movements are clearly distinguishable for Subjects 1, 4, and 5. However, for Subjects 2 and 3, the active locations of two finger movements are almost indistinguishable.

**Fig 11:**
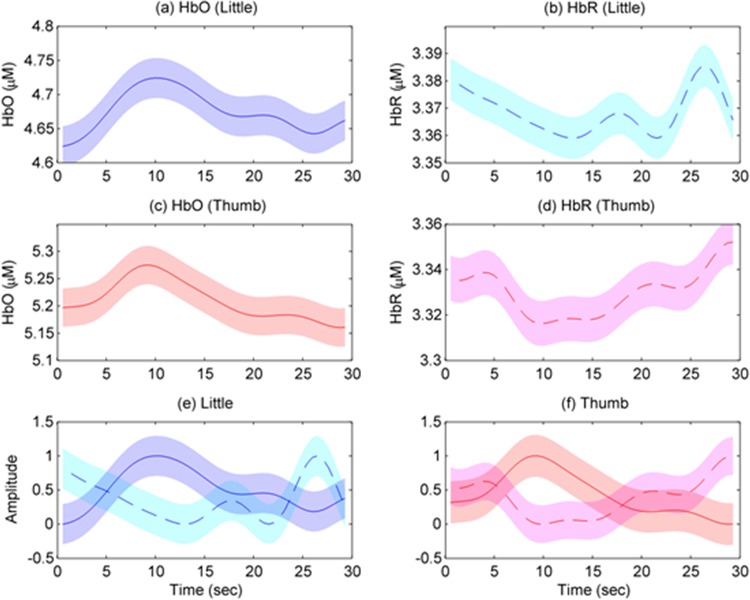
Averaged HbOs and HbRs of little and thumb finger movements (Subject 4). (a)/(b) present the averaged HbO/HbR of little finger movements, (c)/(d) depict the averaged HbO/HbR of thumb movements, (e)/(f) plot the HbO and HbR of little/thumb finger movements (standardized in the 0~1 range) from (a)-(b)/(c)-(d), and the boundaries denote one standard deviation.

**Fig 12:**
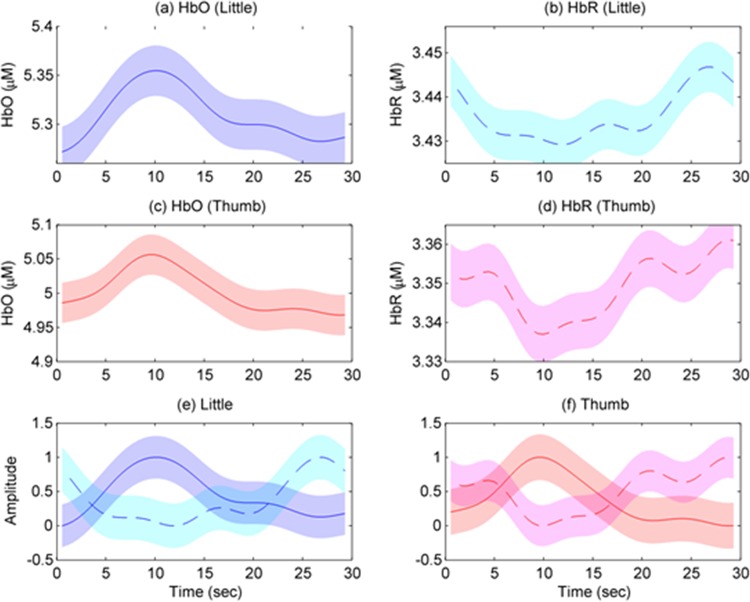
HbOs and HbRs of little and thumb finger movements averaged across five subjects (see S1 Dataset for more details). (a)/(b) present the averaged HbO/HbR of little finger movements, (c)/(d) plot the averaged HbO/HbR of thumb movements, (e)/(f) plot the HbO and HbR of little/thumb finger movements (standardized in the 0~1 range) from (a)-(b)/(c)-(d), and the boundaries denote one standard deviation.

To assess the representative HR for each finger movement, the HbXs were averaged across all active channels within the ROIs (i.e., *t*-value > *t*_crt_). Fig 11 describes the averaged HbOs and HbRs of little and thumb finger movements (Subject 4). Fig 11(A) and 11(B) plot the averaged HbO/HbR of little finger movements, respectively. Fig 11(C) and 11(D) illustrate the averaged HbO/HbR of thumb movements, respectively. Fig 12 presents the averaged HRs of little and thumb finger movements averaged across five subjects. Fig 12(A) and 12(B) show the averaged HbO/HbR of little finger movements, respectively. Fig 12(C) and 12(D) picture the averaged HbO/HbR of thumb movements, respectively. As can be seen, at the onset time, the starting HbO values of little and thumb finger movements (see Fig 12(A)–12(C)) are 5.27 μM and 4.97 μM, respectively whereas those for HbRs are 3.44 μM and 3.35 μM (see Fig 12(B)–12(D)). It is noteworthy that the mean, peak, time-to-peak values of little finger movements are higher than those of thumb movements (see Table 1). However, the skewness of little finger movements is smaller than that of thumb movements. Assuming that the HRs of little and thumb finger movements are the same. The two-sample *t*-test (i.e., the “ttest2.m” function is available in Matlab) was used to test our hypothesis. The experimental results demonstrated that the HR of little and thumb finger movements are different (*p*-value < 0.05).

**Table 1:** Comparison of parameters of HRs of little and thumb finger movements.

Parameters of HR	Thumb	Little	Average
Mean values (μM)	5.04	5.34	5.19 ± 0.21
Peak values (μM)	5.06	5.35	5.21 ± 0.21
Time-to-peaks (sec)	9.39	9.94	9.67 ± 0.39
Skewness	0.57	0.48	0.53 ± 0.06

## Discussion

In this work, a bundled-optode method based on the SRS principle was developed to measure the absolute concentrations of the HbXs upon two finger movements. It is noteworthy that multiple pairs of short- and long-separation channels were utilized in our proposed method for superficial noise removal [28,29]. In the fNIRS area, there is no gold standard to define a short- and/or long-separation detectors yet. However, several researchers demonstrated that the short-separation detector with distance 0.5~1.0 cm could measure the systemic hemodynamic fluctuations from the superficial layers [6,28,29]. In our current work, the short-separation detectors were placed at 0.5 cm to measure the superficial noise. The relative distance for long-separation detectors is normally arranged ~ 3 cm [29]. For our bundled-optode configuration, the long-separation detectors arranged with different distances (1.5, 2.5, 3.0, 3.5, and 4.0 cm) could detect both superficial noises and the brain activities in the different depths. By using (5) in our proposed method, the superficial noises were reduced. Additionally, the patterns in local active brain regions could be precisely detected. Our experimental results demonstrated that the proposed approach can effectively reduce the superficial noise (see Figs 8–10). The CNRs of HbOs computed by using our proposed method were greater than the CNRs of those using the conventional MBLL approach. Furthermore, our proposed method was applied to detect the HRs of little and thumb finger movements and identified their highly active territories as well. In some recent works, the active locations of individual finger movements were determined by using the center of mass, according to which voxel clusters greater than 4 mm^2^ were extracted for each finger [37,42]. In our work, the ROIs were identified and plotted in the 3D space in order to present the highly active territories upon individual finger movements. According to our experimental results, the highly active locations upon the movements of the little and thumb fingers were distinguishable. The active locations upon the thumb movements are in the lateral aspect, while those upon the little finger movements are in the medial aspect (see Fig 5, Subjects 1, 4, and 5). Additionally, the highly active locations of little finger movements are deeper than those of thumb movements (see Fig 5, Subjects 1 and 4). Our obtained results are quite consistent with the relevant previous studies [35,37,42,44]. Even though the obtained results are preliminary, they will certainly contribute to the development of future brain-computer interface applications using non-invasive technique [61–69]. For example, the movements of the different fingers could be used to formulate commands for control of external devices such as robot arms or wheelchairs [70,71]. Moreover, the method could be extended to 3D fNIRS imaging based on each local brain region.

Reiterating the experimental results, the HRs of little and thumb finger movements are different. Table 1 depicts the parameters of the HRs of two fingers. The mean, peak, and time-to-peak values of the HRs of little finger movements are 5.34 μM, 5.35 μM, and 9.94 sec, respectively, which are quite higher than those of the HRs of thumb movements (i.e., 5.04 μM, 5.06 μM, and 9.39 sec, respectively). However, their skewness values are no significant difference (0.53 ± 0.06).

For Subjects 2 and 3, the active territories of the individual finger movements were indistinguishable (see Fig 5). This phenomenon, which had emerged in some of the relevant previous work [36–40], can be explained in two ways: i) the muscle motions of each finger are affected by the others in the same hand; ii) the spatial resolution of our configuration is not yet sufficiently perfect to detect these overlapping regions.

Finally, the current work has certain limitations. Our bundled-optode arrangement comprises 32 optodes that can only record the brain activity in local brain regions with area 1.5 cm × 4 cm. Moreover, we only investigated the active territories of little and thumb finger movements. The active locations of the index, middle and ring finger movements in the same hand were not checked. In future work, we will design a bundled-optode configuration with a larger area and conduct experiments with five-finger movements for the same hand.

## Conclusions

In this paper, the bundled-optode method for measuring the absolute concentrations of HRs and identifying the active territories upon two finger (i.e., little and thumb) movements was presented. By identifying the ROIs and plotting them in the 3D space, the active territories upon the two finger movements were distinguishable. Our results showed that the highly active locations of the little and thumb finger movements are different. Additionally, the mean, peak and time-to-peak values of the HRs in active brain regions of little finger movements were higher than those of the HRs of thumb movements, whereas their skewness values were not significantly different. For our future work, further validation is required for all fingers on the same hand and a large subject group.

## Supporting Information

S1 Dataset The data set of little and thumb finger movements (averaged over 5 subjects). For further understanding our study, recorded fNIRS data were provided as supporting information.
(XLSX)
